# Speech emotion recognition using fine-tuned Wav2vec2.0 and neural controlled differential equations classifier

**DOI:** 10.1371/journal.pone.0318297

**Published:** 2025-02-20

**Authors:** Ni Wang, Danyu Yang

**Affiliations:** 1 College of Mathematics and Statistics, Chongqing University, Chongqing, China; University of Auckland, NEW ZEALAND

## Abstract

Speech emotion recognition (SER) has always been a popular yet challenging task with broad applications in areas such as social media communication and medical diagnostics. Due to the characteristics of speech emotion recognition dataset, which often have small data volumes and high complexity, effectively integrating and modeling audio data remains a significant challenge in this field. To address this, we propose a model architecture that combines fine-tuned Wave2vec2.0 with Neural Controlled Differential Equations (NCDEs): First, we use a fine-tuned Wav2vec2.0 to extract rich contextual information. Then we model the high-dimensional time series feature set using a Neural Controlled Differential Equation classifier. We set the vector field as an MLP and update the model’s hidden state by solving the controlled differential equation. We conducted speech emotion recognition experiments on the IEMOCAP dataset. The experiments show that our model achieves the weighted accuracy of 73.37% and the unweighted accuracy of 74.18%. Additionally, our model converges very quickly, reaching a good accuracy after just one epoch of training. Furthermore, our model exhibits excellent stability. The standard deviation of weighted accuracy (WA) is 0.45% and the standard deviation of unweighted accuracy (UA) is 0.39%.

## Introduction

In the field of artificial intelligence, emotion recognition has emerged as a crucial research direction within natural language processing and computer vision. This technology has increasingly demonstrated its vast potential applications in systems related to human-computer interaction, emotion monitoring, and intelligent services[1]. Among these technologies, speech-based emotion recognition stands out as a significant branch. Due to its ability to intuitively and naturally understand and respond to human emotions, it has broad application prospects in various fields such as social media analysis, intelligent customer service, and assistive diagnosis[2].

The key to speech emotion recognition (SER) lies in extracting data features from limited, small-scale datasets to represent complex emotional expressions [3]. Currently, research can be conducted from two main aspects: acoustic features and linguistic content. Utilizing deep learning for text processing of speech has become a relatively mature research field[4] . Although speech-to-text technology has advanced considerably, using textual content alone for SER is not yet an effective solution. This is because it loses many fundamental acoustic features that express emotions, such as pitch frequency, volume, and formants[5]. Another critical aspect is that temporal information in speech plays an important role in emotion recognition. Temporal information refers to the changes in the speech signal over time, including dynamic features such as rhythm variations and pitch fluctuations, which can help models capture the subtle changes in emotional expression that might not be as apparent in static acoustic features. Therefore, traditional methods of extracting Low-level descriptors (LLDs) followed by manual operations such as statistical pooling layers have not yielded satisfactory experimental results[6]. Consequently, directly extracting time-series data from the speech signal is a better approach to address existing challenges.

Currently, there are many methods for directly extracting features from speech, such as spectrograms, Mel-spectrograms, and MFCCs[7–9]. However, traditional extraction methods rely on intricate feature engineering, requiring manual design and selection of speech features. Wav2vec2.0[10], an advanced end-to-end speech representation model based on Transformers, learns rich representations from large-scale unlabeled speech data through pre-training. These features not only encompass the physical attributes of speech but also implicitly contain information about the speaker’s emotional state. As a result, wav2vec2.0 has demonstrated excellent performance in tasks such as speech recognition[11] and speech emotion recognition[12]. [13,14] have also proposed some fine-tuned models based on wav2vec, achieving good results in emotion recognition tasks. Recently, [15,16] have used the representations extracted by the model as features for emotion recognition tasks. [17] achieved the UA of 74.3% by data augmentation and using fine-tuned wav2vec2.0 model. We replicate their model by replacing the downstream model by Average-Pooling in the time dimension and the UA is 71.7% and the WA is 71.96%. This may be because we did not use any data augmentation operations and directly trained with the features extracted by the upstream model. However, data augmentation operations usually consume resources and manpower. Our end-to-end operation is more portable.

To effectively model time-series data for SER, various research methods have been proposed. [18,19] introduced Hidden Markov Models; [20,21] utilized Support Vector Machines. Additionally, various deep neural network models have been applied to this task, yielding outstanding experimental results [22–24]. On the IEMOCAP dataset, [22] proposed a novel weighted time-pooling strategy and achieved 63.5% WA and 58.8% UA. [23] uses a combined convolution-LSTM higher-complexity model to achieve 68.8% WA and 59.4% UA. [24] achieves 70.6% WA by TDNN-LSTM model. The Neural Control Difference Equations (NCDEs) model[25], a novel deep learning approach, is particularly suitable for handling data with continuous time-series characteristics, such as speech signals. The core idea of NCDEs is to model the evolution of dynamic systems through controlled differential equations. By combining the high-dimensional features extracted by fine-tuned wav2vec2.0 with the dynamic modeling capabilities of the NCDEs model, our work achieves state of art accuracy in SER.

The main contributions of this paper are as follows: (1) We propose a novel model architecture that combines fine-tuned Wav2vec2.0 with Neural Controlled Differential Equations (NCDE) for speech emotion recognition. Compared to other similar baseline models, the NCDEs model demonstrates significant advantages in SER on the IEMOCAP dataset, including high accuracy, exceptional stability, and fast convergence. (2) The NCDEs model can automatically learn the dynamic changes in speech signals without the need for manual design or selection of specific features, thus avoiding human intervention. This is particularly valuable for handling highly complex and nuanced emotional expressions. Our code is available at https://github.com/Ni-W/NCDE-wav2vec2.

## 1 Related work

### 1.1 Fine-tuned wav2vec2.0 model

Wav2vec2.0[10], developed by Facebook AI Research, builds on the original wav2vec model by introducing a more complex self-supervised mechanism. This allows the model to learn speech representations without labeled data by predicting missing parts of the input sequence. By incorporating the Transformer mechanism, the end-to-end wav2vec2.0 model avoids the need for manual feature design and selection, enabling it to more effectively capture subtle variations in speech data, thereby improving the accuracy of speech recognition and processing. The wav2vec2.0 model has demonstrated excellent performance in tasks such as automatic speech recognition and speaker verification[10,12,26,27].

Recently, pre-trained wav2vec2.0 models are fine-tuned on target datasets and then used as feature extractors to obtain features better suited for specific tasks[28,29]. Inspired by this approach, we choose to use the Fine-Tuned wav2vec2.0 model proposed by Li-Wei Chen et al.[17]: P-TAPT (Pseudo-label Task Adaptive Pretraining Model) as the upstream model to extract features suitable for SER. P-TAPT fine-tunes the model by adding a position-wise linear head and running a k-means clustering algorithm, overcoming the limitations of fixed frequency perception scales and resolutions. This approach provides more descriptive and higher-level feature representations, enhancing the model’s ability to capture rapidly changing signals and detailed features. It has achieved good experimental accuracy on the IEMOCAP datasets. The features extracted by P-TAPT contain rich contextual information and emotional representations, making them very valuable for understanding language content and emotion recognition.

### 1.2 Neural controlled differential equations

Neural Controlled Differential Equations (NCDEs), an innovative mathematical tool in modern data science, was first proposed by Patrick Kidger et al. in 2020[25]. NCDEs are commonly used to address issues related to irregularly sampled data. For the experiments in this study on speech emotion recognition, while audio recordings generally have a fixed sampling rate, speech signals exhibit rich dynamic variations along the time dimension. The advantage of NCDEs lies in their ability to capture the intrinsic dynamic properties of signals, such as pitch fluctuations and tempo variations. This enables the model to extract hidden, high-level information within speech features, even under fixed sampling conditions, thereby facilitating emotion recognition. On one hand, controlled differential equations (CDEs) introduce the concept of control paths to ordinary differential equations (ODEs), providing a mathematical description of external interventions in the system. NCDEs extend this framework by integrating neural networks, enhancing the model’s ability to approximate the complex dynamics of nonlinear systems. On the other hand, rough path theory supports the universality of NCDEs. Rough Path Theory[30], proposed by Terry Lyons in 1998, is a method for handling paths with irregularities and high fluctuations, such as financial data[31] and experimental physics data[32]. By enriching the path information, it enables the definition of integrals on more irregular paths. This theory studies highly irregular paths, such as paths without bounded variation.

NCDEs can be viewed as a continuous generalization of RNN models, interpreting input streams as evolving in continuous time. This allows NCDEs to exhibit outstanding advantages in handling complex time-series data, as demonstrated on datasets like CharacterTrajectories[33] and PhysioNet 2019 Challenge[34]. In [25], NCDEs substantially improved convergence speed and model accuracy in speech command recognition tasks[35]. NCDEs naturally model the temporal dependencies of data, enabling the model to capture more subtle changes. Therefore, compared to traditional mathematical models and machine learning methods, NCDEs are more efficient and accurate by handling continuous time-series data.

## 2 Proposed approach

**Fig 1:**
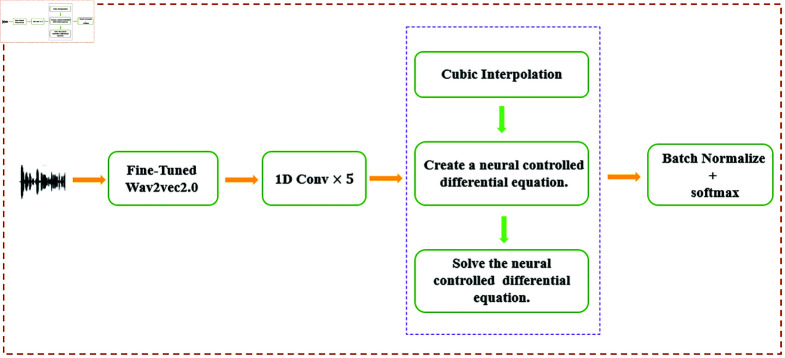
The architecture of NCDEs Classifier.

In this section, we first introduce the relevant definitions and theoretical foundations of NCDEs. Next, we present the neural network model we have constructed. The overall design of the model is shown in Fig 1.

### 2.1 Definition of NCDEs

We first review the definition of the solution to controlled differential equations (CDEs). Let *T* > 0 and let *v* , *w* ∈ *ℕ* .  Let $x:[0,T]\to {\mathbb{R}}^{v}$ be a continuous path of bounded variation. Let $f:{\mathbb{R}}^{w}\to {\mathbb{R}}^{w\times v}$ be Lipschitz continuous. Let $y_{0}\in {\mathbb{R}}^{w}.$

A continuous path $y:[0,T]\to {\mathbb{R}}^{w}$ is said to solve a *controlled differential equation*, controlled by *x*, if


$$y(0)=y_{0},\;y(t)=y(0)+\int_{0}^{t}f(y(s))dx(s)\text{ for }t\in (0,T].$$

Let $f_{\theta }:{\mathbb{R}}^{w}\to {\mathbb{R}}^{w\times v}$ be a neural network depending on parameters *θ* .  Let $\ell_{\theta }^{1}:{\mathbb{R}}^{v}\to {\mathbb{R}}^{w}$ be a neural network depending on parameters *θ* .  And $\ell_{\theta }^{2}$ is a neural network that maps the terminal value of the solution to the target space. Neural networks are typically composed of Lipschitz functions, so a neural network satisfies the Lipschitz condition. Both $f_{\theta }$, $\ell_{\theta }^{1}$ and $\ell_{\theta }^{2}$ will often be parameterised as MLPs.

Define a *Neural Controlled Differential Equation*:


$$y(0)=\ell_{\theta }^{1}(x(0))\;y(t)=y(0)+\int_{0}^{t}f_{\theta }(y(s))dx(s)\text{ for }t\in (0,T]\;z=\ell_{\theta }^{2}(y(T)).$$

The hidden state *y* varies according to *x*, which is very similar to an RNN.

In the NCDEs model, the input data stream serves as the driving path, and the output is an image of the solution to the controlled differential equation.

### 2.2 Universal approximation with NCDEs

Based on Theorem C.25 in Appendix C.2.1 of [36], CDE can represent general functions on data streams. Accordingly, the NCDEs model also possesses universality.

*Any continuous function on a compact subset of the set of continuous bounded variation paths can be uniformly approximated by the output of the NCDEs model.*

In brief, given a compact set of data streams, any continuous function on this compact set can be uniformly approximated by NCDEs under the topology of uniform convergence. The proof of this theorem relies on the boundedness of interpolation and the definition of the topology of time series, as well as the classical neural network and signature universality approximation theorem. This theorem theoretically ensures that the NCDEs model is a universal approximator. Therefore, the functions approximated by NCDEs are sufficiently rich.

### 2.3 NCDEs classifier

NCDEs Classifier is conducted in JAX. JAX can directly leverage the XLA (Accelerated Linear Algebra) compiler to optimize code into efficient low-level instructions, providing a high-performance and user-friendly platform for deep learning tasks. The goal is to achieve more accurate predictions for speech emotion recognition tasks. The construction of the model is as follows:

#### 2.3.1 Transposed convolution module

Using the FT-wav2vec2.0 model, audio files are converted into data streams. Although we have extracted rich feature information, the feature dimension is as high as 768. Directly using this dimension for training would waste expensive computational resources and could lead to memory insufficiency. Furthermore, experiments have shown that reducing the feature dimension before training can significantly shorten training time (by nearly six times for NCDEs and nearly one time for other baseline models). Therefore, due to limited computational resources and with the aim of saving time, reducing computational complexity, and minimizing the risk of overfitting, we reduce the feature dimension for all models using a 1D ConvTranspose layer before training. This operation can improve the accuracy of NCDE classifier. Through convolution operations, the network can capture local patterns and correlations in the input data, retaining important information in the data stream to achieve a more efficient and practical model.

#### 2.3.2 NCDE module

First, a continuous control path is constructed using Cubic Interpolation[37]. This interpolation method meets the theoretical requirements for the universal approximation capability of NCDEs, and enables our solver to solve quickly. Second, an MLP transforms the data into the initial hidden state $y_{0}$ of the CDEs. The MLP effectively captures the nonlinear characteristics of the input data, providing a starting point for subsequent dynamic evolution. Third, the core of this module is a neural vector field $f_{\theta }$, which consists of several MLP with dropout layers. $f_{\theta }$ updates the model’s state at each time step. The linear layers transform data to a high-dimensional space of dimension 220, while the Dropout layers effectively prevent overfitting, ensuring the model has good generalization ability. Finally, the model uses a differential equation solver from the JAX Diffrax library to solve the controlled differential equation.

#### 2.3.3 Output module

A linear layer maps the terminal value of the solution of the differential equation to the prediction space, and then a Softmax activation function is used to output the probability distribution for classes. Finally, batch normalization is applied to further enhance the stability of the training process.

## 3 Experiments and analysis

### 3.1 Data

The IEMOCAP dataset, produced by the University of Southern California, is an interactive emotional motion capture (USC-IEMOCAP) database[38]. This dataset includes a total of five sessions, each containing dialogues between two individuals along with their corresponding labeled speech transcripts (at the phoneme and word levels). We did not use transcripts for training, only speech data. This saves the step of manual text operation, greatly saving manpower and material resources. Each session includes both male and female voices to eliminate gender bias. The IEMOCAP dataset is divided into scripted and improvised dialogues, effectively minimizing the impact of the speakers’ own emotions on the speech. We used all the speech files for training, which is a common approach[39,40]. The dataset comprises nine emotion categories, but based on previous work in SER[6,17], this experiment only considers four emotion categories: Angry, Happy and excited, Neutral, and Sad. Here, Happy/excited are treated as a single category. The distribution of these categories in the dataset is as follows: Neutral (48.8%), Happy/Excited (12.3%), Sad (26.9%), and Angry (12%).

### 3.2 Feature extraction

Li-Wei Chen et al.[17] fine-tuned wav2vec2.0 specifically for the task of speech emotion recognition on the IEMOCAP dataset, resulting in richer feature representations and substantially improved model performance. We use the upstream part of the fine-tuned wav2vec2.0 model, P-TAPT, as the feature extractor. The model and parameters are obtained from [17]. For the IEMOCAP dataset, we extract speech features by segment, with a selected time dimension of 256. For speech segments shorter than 256, we pad with zeros, and for those longer than 256, we extract a central segment of length 256. As a result, only 16.1% of the data was unused in the experiment. Typically, the middle segment of the audio contains more informative content. Thus, we obtained a standardized dataset across the time dimension for training. We extracted 5531 feature segments from the five sessions. For validation, we select one session at a time while using the remaining four sessions for training, performing five-fold cross validation.

### 3.3 Experimental setup

For each model in the experiment, we chose to end training after a uniform epoch count of 15. The hyperparameters of each model were optimized individually. Cross-entropy loss was selected for model optimization. Common metrics for evaluating the performance of classification models were chosen: weighted accuracy (WA) and unweighted accuracy (UA). For each Session, the experiment was repeated five times, and the average accuracy and standard deviation are reported.

We selected a series of neural network models related to NCDEs as baselines, including GRU-*Δ*t[25], GRU-D[41], ODE-RNN[42], SigModel [43] and Average-Pooling[17]. Among these, GRU-*Δ*t, GRU-D, and ODE-RNN were proposed by [25] as baseline models for comparison with NCDEs. GRU-*Δ*t is a GRU with the time difference between observations additionally used as an input; GRU-D builds on GRU-*Δ*t by incorporating learned exponential decay between observations to refine the model; ODE-RNN is a special case of GRU-*Δ*t, applying learned Neural Ordinary Differential Equations [36] to the hidden state between observations. Both SigModel and NCDEs are theoretically based on rough path theory. SigModel calculates the third-order signature to capture higher-order features of the data. Then MLP is used for classification. We refer to this model architecture as SigModel. The signature, as a core concept of rough path theory, is composed of the iterated integrals of a path and is a special case of NCDEs. We implemented this model using the signatory library in PyTorch. At the same time, we replicate the downstream model of [17] within our framework. We refer to this model as the Average-Pooling model. In this model, we directly average the features extracted by P-TAPT across the time dimension and then classify using MLP. Possibly due to the fact that we do not use any data augmentation techniques mentioned in the [17] within our framework, the accuracy obtained by replicating this model is 71.96% (WA) and 71.70% (UA). For our model, after extracting data features using the upstream model in [17], we replace the downstream model with the NCDEs model and get 73.37% (WA) and 74.19% (UA). Moreover, our model is more stable and has a faster convergence speed.

### 3.4 Parameter selection

The choice of hyperparameters is crucial for model training. We took the speech command recognition experiment in [25] as a reference and made grid search to determine the optimal model parameters. The batch size was initially set at 256 and then gradually decreased (128, 64, 32) for experimentation; the dropout rate started from 0.4 and was decreased by 0.05 each time (0.35, 0.3, 0.25, 0.2) for testing; the hidden layer size started from 40 and was adjusted (20, 80, 160). For the learning rate, we also tested a series of different values: 0.001, 0.002, 0.005, 0.01, 0.016, etc. We found that a lower learning rate could achieve a higher classification accuracy. Although compared with a higher learning rate, the training time for one round would increase slightly, the accuracy of our model would increase by 1% - 2%. At the same time, in terms of model stability, the lower learning rate also showed certain advantages. Through different experimental comparisons, we determined the current choice of hyperparameters: batch size [32], dropout rate [0.3], hidden layer size [40], and learning rate [0.001].

### 3.5 The impact of data class imbalance

**Fig 2:**
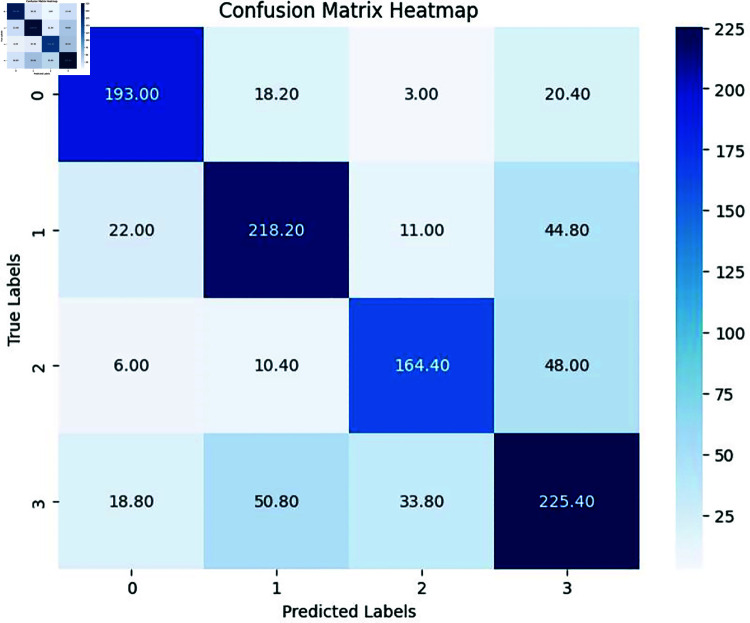
The Confusion Matrix Heatmap of NCDE.

By observing the confusion matrix heatmap in Fig 2, we can clearly see that the IEMOCAP dataset exhibits class imbalance. Class 3 (neutral) has the highest number of correctly classified samples [225.40] (It is the average value of five experiments.) and relatively few misclassified samples, indicating that the model performs best in classifying this category. This is likely because Class 3 (neutral) has the largest proportion of samples, making the model more inclined to learn features of this class during training. In contrast, Class 2 (sad) has fewer correctly classified samples [164.40] and a significant number of samples misclassified as Class 3 (neutral) [48.00]. This may be due to the smaller sample size of Class 2 (sad), which makes it more challenging for the model to extract sufficient features. The imbalance reflected in the confusion matrix heatmap aligns with the characteristics of the IEMOCAP dataset. This demonstrates that class imbalance indeed poses challenges for model training. During training, the model tends to prioritize learning features from majority classes, leading to a tendency to misclassify samples from other classes into these majority classes during testing. Additionally, the loss contribution from majority class dominates the overall loss, while the loss from minority class samples contributes less to gradient updates. As a result, the model struggles to optimally adjust its parameters for minority classes, reducing classification accuracy for these categories and limiting the model’s generalization ability. This phenomenon highlights one of the reasons why training models on the IEMOCAP dataset remains a challenge.

### 3.6 Results and discussion

As can be seen from Table 1, for the two accuracies of WA and UA, our model has achieved the highest accuracy among all experimental models, which are 73.37% and 74.19% respectively. Compared with the average pooling operation in the time dimension, it is improved by 1.41% and 2.49% respectively. In addition, our model is very stable, with standard deviations of 0.45% and 0.39% respectively, which are the smallest among all models. At the same time, SigModel also has good stability. As a special case of NCDEs, Signature has a standard deviation of less than 1%. This shows that models under the rough path theory can indeed learn rich context features and obtain more stable models. This may be because the model based on rough path theory considers the continuous evolution of the speech signal over time and the hidden space variable also changes with the input over time, making the model insensitive to the small perturbations commonly found in the data and enabling it to adapt more smoothly to different emotional expressions, which leads to a significant improvement in stability.

**Table 1:** Comparison of models with WA and UA(%).

Model	WA	UA
GRU-*Δ*t	56.94%±5.43%	55.76%±5.22%
GRU-D	59.75%±7.36%	62.72%±4.18%
ODE-RNN	61.89%±4.20%	65.38%±3.51%
SigModel	71.06%±0.51%	71.55%±0.85%
Average-Pooling	71.96%±2.18%	71.70%±3.31%
NCDEs-Classifier (ours)	**73.37% ± 0.45%**	**74.19% ± 0.39%**

**Fig 3:**
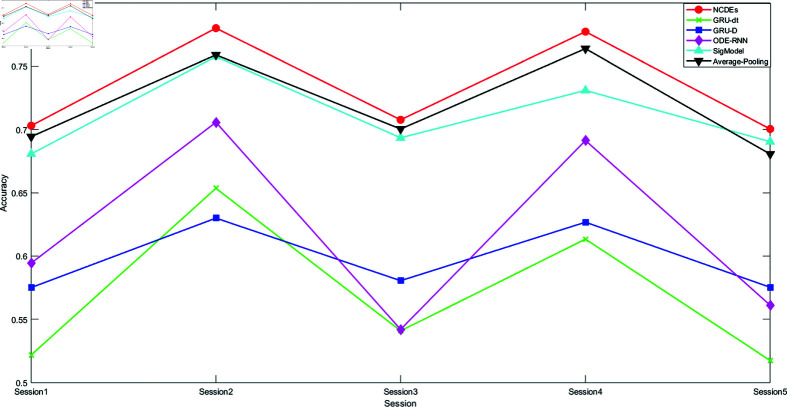
A line chart of the WA accuracy achieved by each baseline model in 5 sessions.

**Fig 4:**
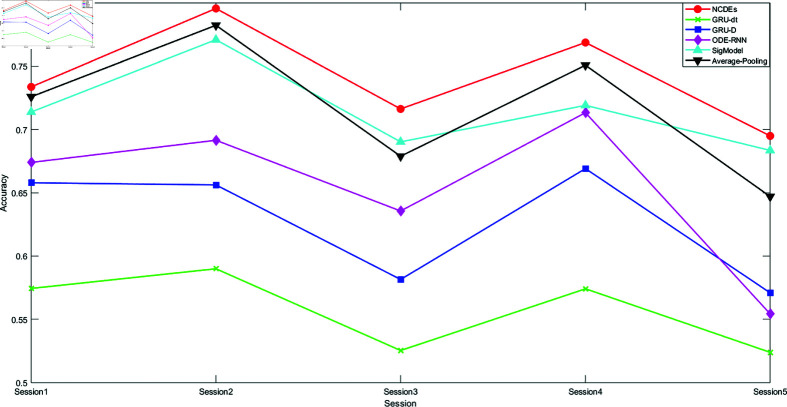
A line chart of the UA accuracy achieved by each baseline model in 5 sessions.

Figs 3 and 4 respectively present the WA and UA accuracies achieved by each model in 5 Sessions. It can be intuitively seen that the NCDEs-Classifier performs the best in each Session. In addition, it is observed that on the Session2 and Session4, the performance of each model is better than that on other Sessions. This may be related to the uneven distribution of the datasets themselves. The number of training sets in these two Sessions is also larger.

**Fig 5:**
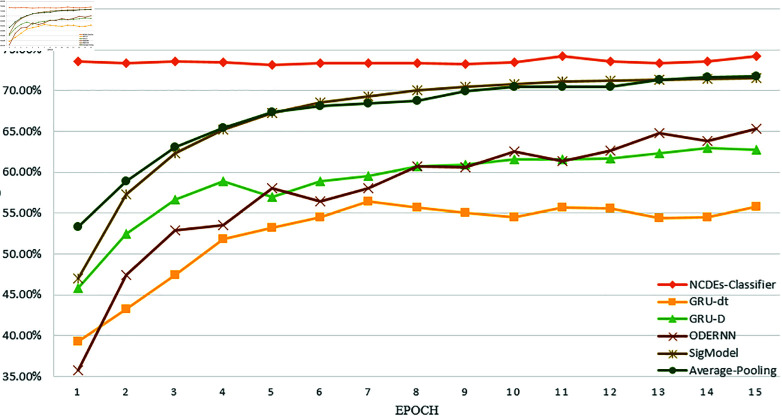
The UA accuracy of each of the first 15 epochs.

Fig 5 shows the UA accuracy of each of the first 15 epochs during the training process. It can be seen that the convergence speed of our model is very fast. In the first epoch, a very high UA accuracy of 73.37% can be achieved, and a high accuracy can be maintained in each subsequent epoch. The situation of WA is similar. The accuracy can reach 72.88% in the first epoch. This enables our model to greatly save computing resources, improve efficiency, and has application value. The accuracies of the other baseline models in the first few rounds are often very low.

Overall, our proposed model, NCDEs Classifier combined with fine-tuned wav2vec2.0, has an outstanding performance in the speech emotion classification task on the IEMOCAP dataset. Our model has not added any data augmentation operations and has good portability. At the same time, the model has high accuracy and a fast convergence speed. The fluctuation of the model is very small, indicating high stability.

## Conclusion

In the task of speech emotion recognition, unlike existing methods, this paper proposes a model based on Neural Controlled Differential Equations under rough path theory. Specifically, we update the model’s hidden state by solving controlled differential equations, which has outstanding advantages in handling complex time-series data. Experiments on the IEMOCAP dataset further demonstrate that our proposed model exhibits superior performance and great practical value. In future work, we plan to implement it on other datasets, such as the Surrey Audio-Visual Expressed Emotion (SAVEE) dataset, to further verify the portability of our model.
